# Alpha-Lipoic Acid as a Nutritive Supplement for Humans and Animals: An Overview of Its Use in Dog Food

**DOI:** 10.3390/ani11051454

**Published:** 2021-05-19

**Authors:** Reshma M. Anthony, Jennifer M. MacLeay, Kathy L. Gross

**Affiliations:** Hill’s Pet Nutrition, Inc., 1035 NE 43rd Street, Topeka, KS 66617, USA; jen_macleay@hillspet.com (J.M.M.); kathy_gross@hillspet.com (K.L.G.)

**Keywords:** alpha-lipoic acid, healthy adult dogs, antioxidant

## Abstract

**Simple Summary:**

A review of human and animal studies involving alpha-lipoic acid supplementation was conducted to determine the utility of alpha-lipoic acid in dog food. The present literature shows that alpha-lipoic acid has utility as a nutritive additive at concentrations of 2.7–4.94 mg/kg body weight/day and improves antioxidant capacity in dogs.

**Abstract:**

Alpha-lipoic acid (a-LA) is used as a nutritive additive in dog food. Therefore, we performed a systematic review of studies published to date in PubMed, Google Scholar, Cochrane Library and MedlinePlus involving alpha-lipoic acid supplementation, which included human clinical trials as well as animal studies, to evaluate its utility as a supplement in foods for healthy, adult dogs. While an upper limit of alpha-lipoic acid intake in humans has not been conclusively determined, the levels for oral intake of a-LA have been better defined in animals, and distinct differences based on species have been described. The maximum tolerated oral dose of a-LA in dogs has been reported as 126 mg/kg body weight and the LD_50_ as 400 to 500 mg/kg body weight. The antioxidant, anti-inflammatory and neuro-protective benefits of alpha-lipoic acid in dogs were observed at concentrations much lower than the maximum tolerated dose or proposed LD_50_. At concentrations of 2.7–4.94 mg/kg body weight/day, alpha-lipoic acid is well tolerated and posed no health risks to dogs while providing improved antioxidant capacity. This review thereby supports the utility of alpha-lipoic acid as an effective nutritive additive in dog food.

## 1. Introduction

Alpha-lipoic acid (often called α-lipoic acid and abbreviated here as a-LA), also known as thioctic acid, is an organosulfur compound, containing two sulfur (thiol) groups. A-LA is available in its oxidized or its reduced form as dihydrolipoic acid (DHLA) [1]. It is naturally occurring and biosynthesized by all living organisms, including humans [1,2]. In its natural form, it is covalently bonded to protein and serves as a co-factor for essential mitochondrial multienzyme complexes involved in amino acid and energy metabolism [1,2]. In addition to its role as an enzyme cofactor, a-LA is involved in several other cellular and molecular functions, including a role as a powerful antioxidant through several different mechanisms: scavenging reactive oxygen and nitrogen species, chelating metals, and contributing to the repair of damaged proteins and lipids [3,4,5]. In addition to endogenous a-LA, there is a growing biomedical interest in exploring the potential therapeutic benefits of exogenous lipoic acid [6].

Reactive oxygen species (ROS) and reactive nitrogen species (RNS) are free radical or non-free radical, highly reactive chemical compounds formed continuously in plants and animals as by-products of aerobic metabolism or in response to stress. Alpha-lipoic acid can effectively scavenge and neutralize a wide range of ROS and RNS such as superoxides, dioxygen species, hydroxyl and peroxyl radicals, peroxynitrite and perhypochlorite [4]. These radicals are known to play a vital role in diseases such as diabetes, cancer and heart disease. When an antioxidant scavenges a ROS or RNS, it gets oxidized itself and cannot scavenge additional reactive species unless it has been reduced. Alpha-lipoic acid plays an important role in the regeneration of other antioxidants such as glutathione, vitamin E, vitamin C, and coenzyme Q10 [2,4,7]. It can regenerate vitamin E from its oxidized form indirectly as well as regenerate ascorbic acid from dehydroascorbic acid [8]. Redox-active metal ions, such as free copper, iron and cadmium, can cause oxidative damage by catalyzing reactions that generate highly reactive species. Alpha-lipoic acid has been shown to directly or indirectly chelate toxic metals [8,9,10,11,12]. It chelates metals indirectly by its ability to increase intracellular glutathione, which in turn functions as a metal chelator [4,8,9,10,11,12]. Metals such as manganese, zinc, cobalt, nickel, lead, cadmium, iron, and copper can form complexes with a-LA and DHLA [4,10,11,12]. Such complexation of metals by a-LA and DHLA may help support antioxidant activity. Formation of complexes with Cu^2+^ explains protection by a-LA in Cu^2+^-induced lipid peroxidation [12]. Similarly, a-LA has been shown to chelate and reduce iron and protect against peroxidation of lipids [13]. A-LA helped prevent Cd^2+^ induced peroxidation of lipids in the heart, brain and testes of rats [14,15,16] and lead-induced testicular toxicity in adult rats [17]. A-LA can also function as an antioxidant by repairing damaged molecules of physiological structures such as lipids, nucleic acids and proteins [4,8].

Alpha-lipoic acid is soluble both in water and lipids and hence can function as an antioxidant inside and outside cells [8]. It can function as an antioxidant essentially in all tissues and is referred to as a universal antioxidant or the antioxidant of antioxidants [7,8,9,10,18]. Because of its potent antioxidant properties, a-LA has been shown to contribute to cardiovascular and cognitive health, anti-aging, detoxification, anti-inflammation, anti-cancer, and neuro-protection [3,8]. Very rare cases of lipoic acid deficiency have been described in human patients by an autosomal recessive pattern of inheritance of mutations in genes coding for proteins of the lipoic acid biosynthetic pathway. These include genes for lipoyl transferase 1 (LIPT1), lipoic acid synthetase (LIAS), and dihydrolipoamide dehydrogenase (DLD, which is the E3 component of the α-ketoacid dehydrogenase complex) [19,20,21]. In such cases, the lack of lipoic acid-dependent enzymatic activity due to a deficiency in synthesizing endogenous lipoic acid cannot be salvaged by administration of exogenous a-LA [19]. Clinical signs include a range of defects depending upon the point in the pathway affected. The most severe symptoms include non-ketotic hyperglycinemia with early-onset convulsions and defects in mitochondrial energy metabolism, encephalopathy and cardiomyopathy [19,20,21].

As a-LA is ubiquitous in nature, it is found in the diets of humans and animals, such as in vegetables, fruits and in meats especially in organ tissues with higher metabolic rates such as the heart, liver and kidneys [22,23]. The endogenous and dietary a-LA is assumed to be sufficient for metabolism. However, it is likely that sufficient amounts of a-LA are not consumed in a standard Western diet. Additional a-LA in food or as a supplement may offer benefits. Reports suggest that a-LA may be conditionally required in older animals [24]. Over the last several decades, the use of a-LA as a dietary supplement in human and veterinary markets has increased and so have concerns about its utility [25]. A systematic review of human and animal studies involving alpha-lipoic acid supplementation was conducted to determine the utility of alpha-lipoic acid supplementation in foods for healthy, adult dogs.

## 2. Methods Used for Systematic Review

This section details the search strategy and the criteria used for selection of studies for this systematic review. The following databases were searched for studies involving alpha-lipoic acid supplementation, which included human clinical trials as well as animal studies to evaluate its utility as a supplement in foods for healthy, adult dogs, published from 1960 until January 2021: PubMed, Google Scholar, Cochrane Library and MedlinePlus. Additionally, a manual search among reference lists of the most relevant articles was conducted to review and reference any articles that were not covered in the databases searches. The authors independently performed the searches to review articles which studied the association between alpha-lipoic acid supplementation and its safety and efficacy in animals and humans. A broad search using the keywords ‘alpha–lipoic acid’ yielded 5462 results and 34,400 results in PubMed and Google Scholar, respectively. To narrow the search, keywords ‘alpha-lipoic acid in animals’ and ‘alpha-lipoic acid in humans’ were used, which narrowed the number of results to 2589 for animals, 2355 for humans in PubMed and 22,200 for animals and 24,400 for humans in Google Scholar. Results from searches in Cochrane Library and Medline Plus were helpful in the review of articles on human studies involving alpha-lipoic acid. Further searches were performed using additional keywords such as alpha-lipoic acid supplementation, benefits of alpha-lipoic acid, safety of alpha-lipoic acid in humans and animals, use of alpha-lipoic acid in animal feeds, use of alpha-lipoic acid in dogs, human clinical trials involving alpha-lipoic acid supplementation, alpha-lipoic acid in veterinary markets, recommended doses of alpha-lipoic acid. More than 200 articles and abstracts that published on alpha-lipoic acid characterization, function and importance of alpha-lipoic acid as well as findings on the safety and beneficial effects of alpha-lipoic acid in humans and animals were reviewed and 139 most relevant articles were referenced. Articles were restricted to those published in the English language. As the use of alpha-lipoic acid has increased in recent years and as the field is rapidly evolving, the authors ensured that this review included approximately one-third of the references published within the last five years.

## 3. Effectiveness of Alpha-Lipoic Acid in Humans

A-LA has been shown to be safe in humans with well-documented tolerability in several human clinical trials [26,27,28,29,30,31]. In lipoic acid studies involving healthy volunteers, 200–600 mg per person daily of lipoic acid was administered either orally or as an intravenous solution and no serious side effects or adverse events were noted [32,33,34,35,36]. In studies on a-LA used to treat diabetic peripheral neuropathy, the compound has been found to be useful. Studies have used intravenous doses of 600 mg/day for three weeks [37] or oral lipoic acid of doses up to 1800 mg/day for seven months [38] or 1200 mg/day for two years [28], and none of them reported serious adverse effects. In a four-year clinical study involving patients with diabetic neuropathy on an oral treatment of 600 mg/day of lipoic acid for four years, there was no significant difference in the number of adverse events and serious adverse events compared to those in the placebo group [39]. In a pilot study involving subjects with multiple sclerosis, oral administration of 2400 mg/day for two weeks was found to be well tolerated [40]. The most common side effects of supplementation with oral lipoic acid were allergic reactions of the skin, such as rashes, itching and hives [40,41]. Nausea, vomiting, abdominal pain, diarrhea and vertigo were also occasionally reported. In one study, the incidence of nausea, vomiting, and vertigo was found to be dose dependent [41]. Two mild cases of anaphylactoid reactions and one severe case of anaphylaxis were reported after intravenous lipoic acid administration [42]. A retrospective observational study showed that daily oral supplementation with 600 mg of lipoic acid between week 10 and week 37 of gestation did not cause any adverse effects in the mothers or their newborn children [43].

In addition to being safe as a dietary supplement in humans, a-LA has been shown to have beneficial effects in several diseases characterized by oxidative stress such as diabetes, multiple sclerosis, and dementia. Chronically high blood glucose concentrations is the hallmark of diabetes which is a serious global health problem. Several studies involving individuals with type 2 diabetes have shown that high doses of a-LA administered orally or intravenously improve glucose utilization, improve insulin sensitivity, lower fasting blood glucose concentrations, insulin concentrations, as well as blood hemoglobin A1c concentrations (hemoglobin A1c represents the average blood glucose over the past three months) [44,45,46,47,48,49]. Approximately half of diabetic patients develop some degree of peripheral neuropathy, which is a type of nerve damage that can cause loss of sensation, pain and weakness, especially in the lower extremities [50]. Peripheral neuropathy is the number one cause of amputation of lower limbs in diabetic patients [51]. Several clinical trials in humans suggest that treatment with oral or intravenous lipoic acid helps reduce symptoms caused by diabetic peripheral neuropathy [37,38,39]. Chronic hyperglycemia may cause damage to blood vessels in the retina, often resulting in diabetic retinopathy [52]. Results from a clinical study showed that daily oral administration of 300 mg of lipoic acid for three months prevented further degradation of contrast sensitivity in patients with diabetes compared to placebo [53]. The study also showed that the administration of the same dose of a-LA improved contrast sensitivity in healthy patients compared to placebo [53]. Lipoic acid is approved for use in the treatment of diabetic neuropathy and retinopathy in Germany for over 50 years and is available by prescription [45,54]. Preliminary clinical trials in humans involving supplementation with lipoic acid showed some improvements in symptoms of multiple sclerosis, cognitive impairment and dementia [40,55] as well as slowing of whole brain atrophy in a two-year study of multiple sclerosis patients [56].

An upper limit for a-LA consumption in humans has not been conclusively established. However, a dosage of 20–50 mg daily is recommended for general antioxidant support [44]. The recommended dosage of a-LA for the treatment of diabetes is 300–600 mg daily. A-LA is available as an over the counter dietary supplement in the United States [45]. Taking lipoic acid with food is known to decrease its bioavailability; hence it is recommended that lipoic acid be taken half an hour to an hour prior to a meal [26,41]. Alpha-lipoic acid is available naturally in foods as the R-isomer and is bound to lysine in proteins [26,57]. Lipoic acid in supplements is available as a racemic mixture of R- and S-lipoic acid (also referred to as *d,l*-lipoic acid) and is not bonded to protein [57]. The amounts of lipoic acid available in dietary supplements vary between 50–600 mg and could be as much as 1000 times higher than the amounts consumed by dietary intake. Lipoic acid in food does not cause detectable increase of free lipoic acid in human plasma or in cells [6,26]. However, high oral doses (≥50 mg) of free lipoic acid increase levels of free lipoic acid in human plasma and cells [26,32]. Human studies have shown that approximately 30–40% of an orally administered racemic mixture of R- and S-lipoic acid is absorbed [26,32]. All published human clinical trials involving a-LA supplementation have used racemic a-LA [32,33,34,35,36,37,38,39,40,41]. Following oral administration with racemic a-LA, the peak concentrations of R-LA in plasma were found to be 40–50% more than those of S-LA, indicating that R-LA may be more efficiently absorbed than S-LA, but both isomers were metabolized and eliminated rapidly [26,58,59]. A study involving 19 healthy adults suggests that the bioavailability of the R and S-isoforms of a-LA may vary with age and gender [60]. Concentrations of alpha-lipoic acid in plasma typically peak within an hour and then decline rapidly [26,32,59]. A-LA is swiftly reduced to DHLA in cells, and in vitro studies show that DHLA is then quickly exported from cells [60].

## 4. Effectiveness of Alpha-Lipoic Acid in Animals

Several metabolism studies [61,62,63] as well as safety studies [64,65,66,67,68] have been performed using a-LA in animals. Unlike in humans, safe levels for the oral intake of a-LA have been well defined in animals, with distinct differences depending on the species. The LD_50_ of oral lipoic acid for rats was shown to be >2000 mg/kg bwt (body weight) [67,68] (Table 1). A four week sub chronic toxicity study in rats showed no signs of toxicity or clinical symptoms when rats were administered 31.6 or 61.9 mg/kg bwt. Hence, the no observed adverse effect level (NOAEL) for rats was considered to be 61.9 mg/kg bwt/day [67]. A long term (2 year) study involving oral a-LA supplementation of 60 mg/kg/day to rats showed no adverse effects and hence a NOAEL of 60 mg/kg bwt/day was established for long-term a-LA supplementation in rats [68]. Mice showed more susceptibility to the toxic effects of a-LA compared to rats. The LD_50_ of oral lipoic acid for mice was 500 mg/kg bwt for mice [4,67]. There are few studies documenting a-LA toxicity in domestic animals [7,8,64,66,69]. The maximum tolerated dose of alpha-lipoic acid in dogs was found to be 126 mg/kg bwt [66] and the proposed LD_50_ of alpha-lipoic acid administered orally in dogs is reported to be between 400 and 500 mg/kg bwt [64]. A case report of two dogs show that clinical symptoms of acute toxicity and death can occur at single oral doses of approximately 190 mg/kg bwt and 210 mg/kg bwt, respectively [7]. In other studies, a single dose of lipoic acid between 10 and 48 mg/kg bwt was administered to dogs either orally or via subcutaneous/intraperitoneal routes [67,69]. No adverse effects were observed in these reports, and in one of the studies, lipoic acid apparently protected the dogs from arsenite toxicity [66,67]. Signs of a-LA toxicity in dogs include ataxia, vomiting, lethargy, weakness, tremors, hypersalivation and seizures [70]. Hepatic and renal failure can also occur [70]. Therapy for a-LA toxicity is symptomatic and supportive. In cats, doses of 30 mg/kg bwt of a-LA were associated with mild acute hepatocellular damage [9]. Oral administration of 60 mg/kg a-LA in cats is associated with signs of acute clinical toxicity [9]. Cats are therefore extremely sensitive to the toxic effects of a-LA compared to humans, dogs, rats and mice. The maximum tolerated dose of a-LA for cats was determined to be 13 mg/kg bwt [9].

Several studies show that a-LA supplementation is beneficial for animals (Table 2). Alpha-lipoic acid supplementation has been shown to enhance glucose metabolism and improve insulin resistance in rats [71,72]. Studies show that A-LA has a protective effect on retinas of experimental mice models with diabetes [73,74] and autoimmune disorders [75]. Alpha-lipoic acid is also found to reduce blood pressure in hypertensive rats [76] and functions as a vasorelaxant [77]. Alpha-lipoic acid has been proven to reduce oxidative stress [78,79,80,81,82,83,84,85], and function as an anti-inflammatory agent in rat and mice models [82,83,86,87,88,89,90]. Several studies in rats and mice show the protective role of a-LA against tissue and organ damage induced by trauma [91,92,93], radiation [94,95], drugs, pesticides and other toxic agents [96,97,98,99,100,101,102,103,104,105,106,107,108]. Studies indicate that a-LA improves neuro-cognitive function in several animal models [82,89,109,110,111,112]. More recent studies show the role of a-LA in alleviating oxidative stress in poultry as well as improving the oxidative stability of poultry meat and meat products [113,114,115,116]. In these studies, poultry feeds were supplemented with a-LA in the range of 50 mg/kg to 500 mg/kg feed. Studies showed that supplementation with a-LA decreased the average feed intake than that of control birds and also had an effect on reducing body weight gain [114,115]. There was a reduction in abdominal fat in the a-LA treatment groups [117]. The antioxidant properties of a-LA combined with the decrease in fat level in the a-LA treatment groups may have resulted in lipid peroxidation prevention, retarding development of off-flavors and improving lipid stability of meat-based products during storage [113,118,119]. Alpha-lipoic acid supplementation also improved growth performance in birds and the quality of poultry meat [117,118,119,120,121,122,123]. A recent study showed the effect of a-LA in improving semen quality in aged roosters [124]. Alpha-lipoic acid has been used as poultry feed supplement since 2001 [125]. Another study showed that a-LA supplementation in diets could improve the average daily gain, feed conversion ratio, anti-oxidative capacity and meat quality of Hainan black goats, a breed of goats domesticated for their delicious meat [126]. Results showed that addition of a-LA to feeds enhanced meat quality by decreasing shear force value and drip loss and increasing meat tenderness [126]. Studies showed that a-LA supplementation in horse feeds of up to 25 mg/kg body weight had no adverse effects in horses [127,128]. These studies also reported a moderate reduction in oxidative stress in horses undergoing light voluntary exercise [127] as well as in those undergoing strenuous exercise [128].

## 5. Use of a-LA as a Nutrient Additive in Dog Food

As in other animals, a-LA supplementation studies indicate that a-LA is beneficial in dogs (Table 3). Alpha-lipoic acid is available as a dietary supplement in the veterinary market for dogs only and a dose of 1–5 mg/kg/day is recommended [131]. Cataract is a significant problem in diabetic dogs, with 75% of animals affected two years after diagnosis [132]. A preliminary study showed that oral supplementation with 2 mg of a-LA/kg bwt/day significantly reduced and delayed lens opacities than those receiving a mixture of ascorbic acid plus tocopherol [133]. Alpha-lipoic acid acts not only as an antioxidant but potentially as an aldose reductase inhibitor [134], thereby reducing sorbitol formation, the main cause of blinding lens opacification in diabetes. Studies also show that supplementation with a-LA and other antioxidants reduces cognitive dysfunction [111] and improves learning [135] in aged canines. Supplementing dog food with a-LA reduced biomarkers known to increase in dogs with osteoarthritis [136].

A pharmacokinetic study of orally administered a-LA in dogs showed that pharmacokinetic parameters of a-LA were influenced by dose and the mode of administration [137]. Absorption of a-LA is reduced when it is used as an ingredient in extruded dog food compared to its absorption of a comparable dose of orally administered a-LA in the form of a capsule, given with or without food [137]. However, the concentrations of a-LA in plasma increased in proportion with the dose, regardless of its administration orally in a capsule form or as an ingredient in dog food [137]. The maximum serum concentrations of a-LA are within the range of values reported for other species [34] as is the time to reach peak concentrations of orally administered a-LA in plasma [26,32,59,137]. The peak concentration of a-LA in plasma is reduced and the time to reach the peak concentration is delayed when a-LA is an ingredient in extruded dog food compared to the peak concentration and the time to reach peak concentration if a-LA is administered orally with or without food, followed by withholding of food for 12 h [137]. Despite differences, overall, the pharmacokinetic parameters were minimally affected by the fed status of the animal when administered in capsule form. Delays in reaching peak concentrations in plasma in dogs fed the extruded foods may be attributable to the complex matrix used to formulate the foods and hence the slower absorption from the gastrointestinal tract [137].

Results of a clinical study to determine the utility of a-LA in adult beagles was published in Zicker et al. in 2002 [129]. In this study, the utility of a-LA in 30 adult beagles was evaluated. The animals were evenly randomized into five groups, each of which was fed one of five foods containing different concentrations of alpha-lipoic acid (0, 150, 1500, 3000 and 4500 ppm) which corresponded to an exposure of alpha-lipoic acid approximately between 0 and 85 mg/kg bwt. These concentrations were much lower than the maximum tolerated dose in dogs of 126 mg/kg bwt [66] and the oral LD_50_ in dogs of 400–500 mg/kg bwt [64]. The dogs were fed one of the five foods (0, 150, 1500, 3000, and 4500 ppm of a-lipoic acid) as their sole and complete source of nutrition for six months. Evaluations included physical examination for overall health, food intake, body weight, hematologic parameters, serum biochemistry, and ratios of reduced glutathione to oxidized glutathione (GSH:GSSG) in lymphocytes. Reduced glutathione (GSH) is a major intracellular, water soluble antioxidant, and its ratio with oxidized glutathione (GSSG) is used as a marker of oxidative stress. Data showed no abnormalities in the hematologic or serum biochemical parameters. No signs of toxicity were reported at any concentration except at the highest concentration (4500 ppm) of a-lipoic acid. At the 4500 ppm alpha-lipoic acid inclusion level, one dog was removed from the study on Day 21 due to leukocytosis and weight loss. Other adverse events were not reported. The weight of this dog stabilized after removal from the study and the leucocytosis resolved over time. All concentrations of a-LA increased the GSH: GSSG ratio in lymphocytes and the greatest improvement was observed at the lowest level of a-lipoic acid (150 ppm of diet). The maximal effect on GSH:GSSG ratio was observed at approximately 2.7 mg/kg bwt which represented a wide margin over doses associated with side effects and had not previously been reported.

An additional six months of data were published by Pateau-Robinson et al. [138] and included a total of 12 months of feeding data compared to the original 6 months published by Zicker et al. in 2002 [129], but antioxidant effects were not further evaluated in months 7–12. Included in this publication were additional statistical analyses, details of the study system, and health endpoints after 12 months of feeding a-LA acid to the five groups of dogs receiving 0, 150, 1500, 3000, or 4500 ppm of food DMB (on a dry matter basis) or approximately 0.31, 2.53, 26.3, 52.9, and 87.7 mg/kg body weight/day, respectively. The groups were uniform with regard to age, body weight, and gender distribution and indicators of health included animal body weights and clinical chemistry from blood analyses which did not differ after 12 months of feeding. Adverse events that occurred during the study were rare and not attributed to the food. This study demonstrated that the addition of alpha-lipoic acid of up to 3000 ppm in dog food did not pose any health risk to healthy adult dogs.

A recent alpha-lipoic acid study [130] was conducted on a much larger population than in the Pateau-Robinson et al. study, and involved 80 dogs (4 study groups of 20 dogs each) and a long baseline period (washout phase) of 15 months followed by a test period of 6 months. During the baseline period, all animals were fed control food with no alpha-lipoic acid. This long washout was done to minimize residual effects of varying intake of other antioxidants such as vitamin E in the population. Monthly serum tocopherol analysis was done during washout until serum concentrations reached a plateau within the normal reference range. The control food contained 0 ppm alpha-lipoic acid and met or exceeded all nutritional requirements for adult dogs as recommended by the Association of American Feed Control Officials [139]. This food was the same as the food used during the test period, except for the addition of varying concentrations of alpha-lipoic acid in the test foods. Following the baseline period, the dogs were randomized to the four foods containing different levels of alpha-lipoic acid (0, 75, 150 and 300 ppm). The average lipoic acid exposure for the different treatments groups was calculated to be 0, 1.20, 2.7, and 4.94 mg/kg body weight/day, respectively.

During the 21 month study, the general health and well-being of the dogs was assessed by serial physical examinations, hematology, and serum biochemistry assessments. Hematology and serum biochemistry values obtained were compared to normal canine reference ranges to help determine general health of the dogs. Other assessments included measurements of body weight (every other week) and food intake (daily). Measurements of reduced glutathione, oxidized glutathione levels, total glutathione levels and the ratio of reduced to oxidized glutathione in both plasma and erythrocyte lysates of all animals were conducted to determine the antioxidant effects of a-LA. The lipoic acid content of the control and test foods were analyzed prior to the beginning of the study and also periodically during the study to confirm that target supplementation levels of alpha-lipoic acid were achieved. Intake of lipoic acid (mg/kg bwt) was calculated by multiplying the mean food intake (g) with the lipoic acid content (ppm; as fed) and then dividing by the animal’s body weight (kg). Animal body weights, clinical chemistry and hematology data from blood analyses among the alpha-lipoic acid treatment groups did not differ after 6 months of feeding a-LA at the concentrations used in the study. The results showed that alpha-lipoic acid, as part of a complete and balanced food, was well tolerated at the concentrations used and increased the endogenous glutathione activity in healthy adult dogs, supporting its use as an antioxidant.

A review of the results from human and animal studies involving alpha-lipoic acid supplementation show that alpha-lipoic acid enhances glucose metabolism, reduces insulin resistance and improves symptoms related to diabetes in both humans and animals. Alpha-lipoic acid supplementation also showed cognitive benefits in humans and animals. It is interesting to note that the daily alpha-lipoic acid doses administered to see these benefits are similar in humans and canines, at or below 15 mg/kg body weight (assuming that human weight is approximately 60–80 kg). However, the daily alpha-lipoic acid concentrations administered in rats in these studies to see these benefits was much higher, up to 100 mg of alpha-lipoic acid per day. The LD_50_ of alpha-lipoic acid is also much higher in rats compared to that in canines (Table 1) which might reflect different metabolism in dogs or humans. Pharmacokinetic studies show that the values of the maximum serum concentrations of alpha-lipoic acid as well as the time to reach peak concentrations of orally administered a-LA in plasma are very similar across species [26,32,34,59,137]. The human and animal studies also showed that alpha-lipoic acid was well tolerated across species and adverse events, if any, were rare.

## 6. Conclusions

A systematic review of human clinical trials and animal studies involving alpha-lipoic acid supplementation was conducted to determine its utility in foods for healthy, adult dogs. The studies show that alpha-lipoic acid was well tolerated in humans and animals and the supplementation of alpha-lipoic acid posed no health risk in humans or animals at the concentrations studied. A review of canine studies supports that a-LA is safe, well tolerated and effective as a nutritive additive in dog food within the range of concentrations used resulting in an exposure of 2.7–4.94 mg/kg body weight/day. The dogs in these studies remained healthy, did not lose or gain weight, or experience adverse events or serious adverse events. At these concentrations, alpha-lipoic acid showed beneficial antioxidant, anti-inflammatory and neuro-protective effects in healthy, non-gestational, non-lactating adult dogs.

## Figures and Tables

**Table 1:** LD_50_, maximum tolerated dose and NOAEL of alpha-lipoic acid in different animal species studied. * NOAEL= no observed adverse effect level; bwt = body weight of the animal.

Species	LD_50_	Maximum Tolerated Dose (If Known)	NOAEL* (If Known)
Rat	>2000 mg/kg bwt	-	60 mg/kg bwt
Mouse	500 mg/kg bwt	-	-
Dog	400–500 mg/kg bwt	126 mg/kg bwt	-
Cat	30 mg/kg bwt	13 mg/kg bwt	-

**Table 2:** Beneficial Effects of alpha-lipoic acid in animals.

Species	Benefits	Reference(s)
Rat	enhances glucose metabolism	[71]
Rat	improves insulin resistance	[72]
Mice	protects retina in disease states	[73,74,75]
Rat	reduces blood pressure	[76]
Rat	functions as a vasorelaxant	[77]
Rat, Mice	reduces oxidative stress	[78,79,80,81,82,83,84,85]
Rat, Mice	reduces inflammation	[82,83,86,87,88,89,90]
Mice	improves memory	[89]
Rat, Mice	protects against organ damage	[91,92,93,94,95,96,97,98,99,100,101,102,103,104,105,106,107,108]
Mice, Rat, Dog	improves neuro-cognitive recognition	[82,89,109,110,111,112]
Poultry	improves oxidative stability and meat quality	[113,114,115,116,117,118,119,120,121,122,123]
Hainan Goats	enhances meat quality and tenderness	[126]
Horses Dog	reduces oxidative stress improves antioxidant capacity	[127,128] [129,130]

**Table 3:** a-LA supplementation studies in canines. bwt = body weight of the animal.

Reference	A-LA Dose Administered to Dogs	Route of Administration	Number of Dogs in Study
[131]	2 mg/kg bwt per day	Oral	30
[133]	11 mg/kg bwt per day	Oral	12
[135]	2.5–25 mg/kg bwt per day	Oral	27
[136]	0–85 mg/kg bwt per day	Oral	30
[137]	0.31–87.7 mg/kg bwt per day	Oral	30
[138]	0–4.94 mg/kg bwt per day	Oral	80

## Data Availability

No new data were created or analyzed in this study. Data sharing is not applicable to this review article.
